# Evidence of microevolution of Salmonella Typhimurium during a series of egg-associated outbreaks linked to a single chicken farm

**DOI:** 10.1186/1471-2164-14-800

**Published:** 2013-11-19

**Authors:** Jane Hawkey, David J Edwards, Karolina Dimovski, Lester Hiley, Helen Billman-Jacobe, Geoff Hogg, Kathryn E Holt

**Affiliations:** 1 Department of Biochemistry and Molecular Biology, Bio21 Molecular Science and Biotechnology Institute, University of Melbourne, Parkville, Victoria 3010, Australia; 2 Department of Agriculture and Food Systems, Melbourne School of Land and Environment, University of Melbourne, Parkville, Victoria 3010, Australia; 3 Microbiological Diagnostic Unit Public Health Laboratory, Department of Microbiology and Immunology, University of Melbourne, Parkville, Victoria 3010, Australia; 4 Department of Health, Coopers Plains, Forensic and Scientific Services, Queensland 4108, Australia

**Keywords:** *Salmonella enterica* serovar Typhimurium (*S*. Typhimurium), Genomics, Microevolution, Outbreak, Whole genome sequencing, Divergence dating, Mutation rate, SNPs, Plasmid, Phage

## Abstract

**Background:**

The bacterium *Salmonella enterica* serovar Typhimurium (*S*. Typhimurium) is one of the most frequent causes of foodborne outbreaks of gastroenteritis. Between 2005–2008 a series of *S*. Typhimurium outbreaks occurred in Tasmania, Australia, that were all traced to eggs originating from a single chicken farm. We sequenced the genomes of 12 isolates linked to these outbreaks, in order to investigate the microevolution of a pathogenic *S*. Typhimurium clone in a natural, spatiotemporally restricted population.

**Results:**

The isolates, which shared a phage type similar to DT135 known locally as 135@ or 135a, formed a clade within the *S*. Typhimurium population with close similarity to the reference genome SL1334 (160 single nucleotide polymorphisms, or SNPs). Ten of the isolates belonged to a single clone (<23 SNPs between isolate pairs) which likely represents the population of *S*. Typhimurium circulating at the chicken farm; the other two were from sporadic cases and were genetically distinct from this clone. Divergence dating indicated that all 12 isolates diverged from a common ancestor in the mid 1990s, and the clone began to diversify in 2003–2004. This clone spilled out into the human population several times between 2005–2008, during which time it continued to accumulate SNPs at a constant rate of 3–5 SNPs per year or 1x10^-6^ substitutions site^-1^ year^-1^, faster than the longer-term (~50 year) rates estimated previously for *S.* Typhimurium. Our data suggest that roughly half of non-synonymous substitutions are rapidly removed from the *S*. Typhimurium population, after which purifying selection is no longer important and the remaining substitutions become fixed in the population. The *S*. Typhimurium 135@ isolates were nearly identical to SL1344 in terms of gene content and virulence plasmids. Their phage contents were close to SL1344, except that they carried a different variant of Gifsy-1, lacked the P2 remnant found in SL1344 and carried a novel P2 phage, P2-Hawk, in place SL1344’s P2 phage SopEϕ. DT135 lacks P2 prophage. Two additional plasmids were identified in the *S*. Typhimurium 135@ isolates, pSTM2 and pSTM7. Both plasmids were IncI1, but phylogenetic analysis of the plasmids and their bacterial hosts shows these plasmids are genetically distinct and result from independent plasmid acquisition events.

**Conclusions:**

This study provides a high-resolution insight into short-term microevolution of the important human pathogen *S.* Typhimurium*.* It indicates that purifying selection occurs rapidly in this population (≤6 years) and then declines, and provides an estimate for the short-term substitution rate. The latter is likely to be more relevant for foodborne outbreak investigation than previous estimates based on longer time scales.

## Background

*Salmonella enterica* serovar Typhimurium (*S*. Typhimurium) is a frequent cause of gastroenteritis in humans [1,2], including foodborne disease outbreaks [3]. A variant of *S*. Typhimurium phage type DT135 - sometimes referred to locally as 135@ or 135a but without official phage type designation - is amongst the most common forms of *S*. Typhimurium in Australia [4,5] and has been isolated from chickens and eggs in Australia [6]. *S*. Typhimurium phage type 135 and 135@ have been associated with multiple foodborne outbreaks in Australia, which are generally epidemiologically linked to the consumption of eggs [7-13] or chicken [14-16].

In 2005, 2007 and 2008 a series of seven outbreaks of *S*. Typhimurium 135@ occurred in Tasmania, Australia [12,13]. These outbreaks involved 193 microbiologically confirmed cases of *S*. Typhimurium 135@ infection, and were each linked to the consumption of raw egg-containing foods through epidemiological investigations conducted at the time [12,13]. For outbreaks 2, 5 and 7, *S*. Typhimurium 135@ was isolated from a food source implicated during the investigation. While different raw egg-containing foods (bakery goods, mayonnaise) and retail outlets (bakeries, cafés and restaurants) were implicated in the various outbreaks, each was traced back to eggs supplied from the same farm [12,13]. *S*. Typhimurium 135@ was isolated from the farm in December 2005 and January 2006, which subsequently ceased to operate.

The series of isolates collected during these outbreaks, linked to a single farm source, provides a unique opportunity to investigate the possible microevolution of a clinically important *S*. Typhimurium clone in a natural but spatiotemporally contained bacterial population. In order to determine the unique features of *S*. Typhimurium 135@, consider its microevolution and explore the utility of whole genome sequencing in understanding intermittent foodborne outbreaks, we sequenced 12 isolates associated with the Tasmanian outbreaks and performed phylogenetic and comparative genomic analysis.

## Results

### Sequencing and phylogenetic analysis

**Figure 1:**
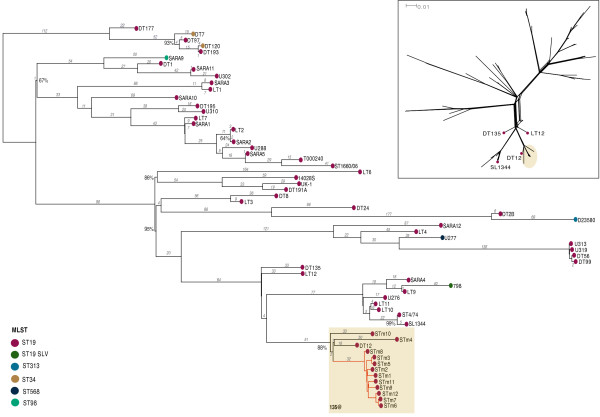
**Phylogenetic tree of *S*. Typhimurium genomes.** Maximum likelihood tree for *S*. Typhimurium based on SNPs identified by mapping to the reference chromosome for *S*. Typhimurium SL1344 (isolate details in Tables 1 and 2), excluding those SNPs identified in phage or repeat regions. Inset shows a neighbour-joining split network of the same SNP data. Shaded region indicates *S*. Typhimurium 135@ isolates sequenced in this study, for which a high resolution tree is given in Figure 2; red branches indicate the farm clone. Bootstrap support is shown for those bipartitions with <100% bootstrap support; tree branches are labelled with the number of SNPs contributing to the branch.

Twelve isolates were selected for sequencing based on their epidemiological link to the Tasmanian outbreaks (including 8/193 isolates from 5 Tasmanian outbreaks, 2/3 farm isolates and 2/100 sporadic isolates) and multi-locus VNTR analysis (MLVA) profiles, such that each outbreak was represented by at least one clinical isolate possessing the dominant MLVA profile for the outbreak; linked food isolates were included where available for that outbreak; and sporadic isolates were those with the closest MLVA profiles to the outbreak isolates. Details of the twelve *S*. Typhiumurium 135@ isolates, named STm1 to STm12 and sequenced via Illumina HiSeq, are given in Table 1. They include seven isolates from 2005/2006 (4 human cases – 1 sporadic, 1 food isolate and 2 farm isolates); two isolates from 2007 (one from an outbreak case and one sporadic case that occurred six months later); and three isolates from the 2008 outbreak (two human cases and a food isolate). The genome assemblies were first reported in [17]. To investigate the relationships between the outbreak isolates, we mapped the sequenced reads to the reference chromosome sequence for *S*. Typhiumurium strain SL1344 (phage type DT44, accession NC_016810.1). Using the single nucleotide polymorphisms (SNPs) identified from mapping, we inferred a maximum likelihood phylogenetic tree. We also included all publicly available *S*. Typhimurium chromosome sequences, including DT135, in the analysis for comparison (Table 2). The tree (Figure 1) confirmed that SL1344 was the closest finished reference to the *S*. Typhimurium 135@ isolates, and therefore the most suitable reference for read mapping, SNP calling and gene content comparison.

**Table 1:** ***S*. Typhimurium isolates sequenced for this study**

**Isolate**	**Year**	**Source (Outbreak)**	**Mean depth**	**Reads mapped**	**Reads accession**	**Contigs (Total bp)**	**Assembly accession**
STm1	2006	Farm A (investigation)	999x	99%	SRR925393	119 (4,944,286)	AMDX02
STm2	2005	Farm A (investigation)	366x	99%	SRR926593	227 (5,019,159)	AMDY01
STm3	2005	Food (OB2)	395x	99%	SRR926594	106 (4,945,185)	AMEB02
STm4	2007	Human case (sporadic)	335x	99%	SRR926595	150 (4,968,080)	AMEC02
STm5	2005	Human case (OB2)	1118x	99%	SRR926602	458 (4,943,760)	AMEH02
STm6	2008	Food (restaurant A, OB7)	302x	99%	SRR926596	84 (4,938,199)	AMED02
STm7	2008	Human case (restaurant A, OB7)	267x	99%	SRR925415	117 (5,090,822)	ATWR01
STm8	2005	Human case (OB1)	311x	99%	SRR926597	102 (4,937,750)	AMDZ02
STm9	2007	Human case (OB6)	350x	99%	SRR926598	90 (4,932,393)	AMEA02
STm10	2005	Human case (sporadic)	523x	99%	SRR926599	104 (4,946,110)	AMEE02
STm11	2005	Human case (OB5)	368x	99%	SRR926600	118 (4,948,677)	AMEF02
STm12	2008	Human case (restaurant A, OB7)	354x	99%	SRR926601	262 (4,990,531)	AMEG02

Isolates studied were numbered STm1, STm2, up to STm12 and were all phage type 135@ (STm135@). OB = outbreak. Mapping statistics refer to mapping against the *S*. Typhimurium SL1344 reference chromosome (mean depth of mapped reads and proportion of total reads mapped to this reference).

**Table 2:** **Publicly available *S*. Typhimurium genomes included in this study**

**Isolate**	**Accession**	**Reference**
14028S	CP001363	[18]
UK-1	CP002614	[19]
D23580	FN424405	[20]
798	CP003386	[21]
ST4/74	CP002487	[22]
SL1344	NC_016810	[20]
LT2	AE006468	[23]
T000240	NC_016860	[24]
ST1660/06	AJTU01	[25]
LT2	ERS007491	[26]
LT6	ERS007492	[26]
LT1	ERS007487	[26]
LT3	ERS007489	[26]
SARA5	ERS007503	[26]
LT4	ERS007490	[26]
LT8	ERS007494	[26]
LT7	ERS007493	[26]
LT9	ERS007495	[26]
LT10	ERS007496	[26]
LT11	ERS007497	[26]
LT12	ERS007498	[26]
SARA1	ERS007499	[26]
SARA2	ERS007500	[26]
SARA3	ERS007501	[26]
SARA4	ERS007502	[26]
SARA9	ERS007507	[26]
SARA10	ERS007508	[26]
SARA11	ERS007509	[26]
SARA12	ERS007510	[26]
DT24	ERS007582	[26]
DT41B	ERS007586	[26]
DT12	ERS007564	[26]
DT120	ERS007566	[26]
DT135	ERS007567	[26]
DT177	ERS007572	[26]
DT191A	ERS007574	[26]
DT193	ERS007576	[26]
DT195	ERS007578	[26]
DT2B	ERS007580	[26]
U302	ERS007606	[26]
U310	ERS007608	[26]
DT56	ERS007588	[26]
DT7	ERS007590	[26]
DT8	ERS007592	[26]
DT97	ERS007594	[26]
DT99	ERS007596	[26]
DT1	ERS007598	[26]
U276	ERS007600	[26]
U277	ERS007602	[26]
U288	ERS007604	[26]
U313	ERS007611	[26]
U319	ERS007613	[26]
DT104	ERS007562	[26]

**Figure 2:**
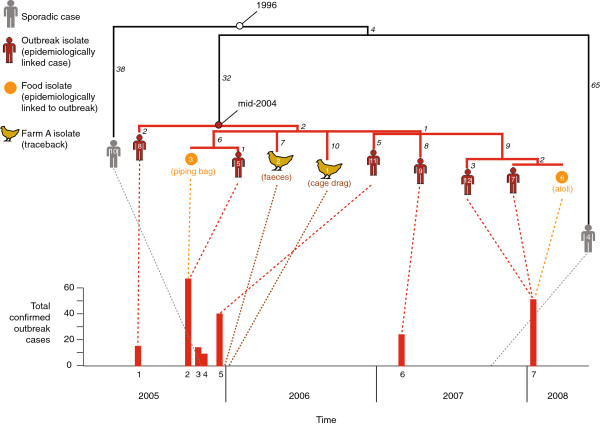
**Phylogeny and timeline of outbreak-related *S*. Typhimurium 135@ isolates.** Maximum likelihood tree of the *S*. Typhimurium 135@ isolates, based on SNPs identified by mapping to the reference chromosome for *S*. Typhimurium SL1344. Bootstrap values for each bipartition were 100%. Isolates are labelled by their STm number and an icon indicating the specimen type (according to the legend provided); black numbers next to branches indicate the number SNPs contributing to that branch. Red bar graph indicates the timing and size of the seven outbreaks. Red branches in the tree indicate the farm clone; dashed lines link each isolate to its time of isolation and outbreak membership.

The phylogenetic tree (Figure 1) showed that the *S*. Typhimurium 135@ isolates formed a clade that was closely related to SL1344 and included a DT12 isolate. This clade (shaded in Figure 1) was separated from SL1344 by just 160 SNPs. Full details of the SNPs that varied within the 135@ clade are provided in Additional file 1: Table S1. Ten of the 12 *S*. Typhimurium 135@ isolates formed a tight clonal group, displaying ≤23 SNPs between pairs of isolates (red branches in Figures 1, 2). The branch leading to this clone was defined by 32 SNPs that the members of the clone share relative to all other *S*. Typhimurium analysed (Figures 1, 2). The *S*. Typhimurium 135@ isolates outside this clone were STm10, isolated during the fourth 2005 outbreak and STm4, isolated from a sporadic case between the 2007 and 2008 outbreaks. The publicly available DT12 genome, isolated from a human infection in the UK in 2009 [26], was equidistant from STm4, STm10 and the *S*. Typhimurium 135@ clonal group (Figure 1). Interestingly, the publicly available DT135 genome, also isolated from a human infection in the UK in 2009, was not within the *S*. Typhimurium 135@ group but clustered near the common ancestor of *S*. Typhimurium 135@ and SL1344.

### Dissecting the series of outbreaks

Figure 2 shows a detailed phylogeny for the *S*. Typhimurium 135@ isolates, linked to a timeline depicting the size and timing of the series of outbreaks as first reported in [12,13]. *S*. Typhimurium 135@ isolated from the same outbreak were near-identical to one another (0–5 SNPs), but were differentiated from other outbreaks by >10 SNPs (Figure 2). The two isolates from the second 2005 outbreak (STm3, STm5) shared 6 SNPs that were not detected in other isolates, and differed from one another at just one SNP. STm5 was from a human case, and STm3 was isolated from a piping bag at the bakery to which the outbreak had been traced [13]. The genomic data therefore provides strong independent evidence that the bacteria isolated from the bakery and the human infection were linked by a short chain of transmission. Similarly, the three isolates from the 2008 outbreak (STm6, STm7, STm12) also clustered closely together, sharing 9 SNPs that were not detected in other isolates (Figure 2). This outbreak was traced to a restaurant [12], from which STm6 was isolated from aioli (mayonnaise made with raw egg). Again the genomic data strongly supports the finding that raw egg-containing food at the restaurant was a source of transmission during the outbreak [12].

The clonal group includes single representative cases from three other outbreaks, and two isolates from the farm to which all outbreaks have been linked (STm1, STm2). Interestingly, while isolates from the same outbreak were nearly identical and shared ≥5 SNPs differentiating them from other outbreaks, isolates from different outbreaks were roughly equidistant from each other and from farm isolates (Figure 2). This suggests that the clonal group to which all outbreak isolates belong (red branches in Figures 1, 2) represents a broader population of *S*. Typhimurium 135@ circulating at the farm, and that this population descends from a single ancestral strain introduced into the farm some time previously. As a corollary, the variation present within the outbreak clone therefore potentially represents the diversification of *S*. Typhimurium 135@ through microevolutionary processes which occurred *in situ* at the farm, whose chicken population presumably provided a reservoir host for the bacteria. We therefore refer to this group, highlighted in red in Figures 1 and 2, as the “farm clone”.

### Dating the emergence of S. Typhimurium 135@ and the farm clone

In order to estimate the likely date of emergence of the farm clone, we analysed the SNP data using Bayesian and maximum likelihood analyses (Table 3). The maximum likelihood tree provided strong evidence for a constant accumulation of SNPs over time within the farm clone (Pearson R^2^ = 0.71), with a substitution rate of 7 × 10^-7^ site^-1^ year^-1^ or 3 SNPs per chromosome per year. The Bayesian and maximum likelihood methods gave similar results (Table 3), supporting a substitution rate of 5-19 × 10^-7^ site^-1^ year^-1^ or 3–5 SNPs per year and indicating the most recent common ancestor (mrca) for the farm clone existed in 2003–2004 (95% HPD, 2002–2005). Consistent with this, STm8 from the first outbreak in June 2005 had acquired only two SNPs since the mrca, whereas the 2008 isolates had acquired 14–15 SNPs since the mrca (Figure 2).

**Table 3:** **Divergence dating analysis for outbreak-related *S. *** **Typhimurium 135@**

	**BEAST**	**Patho-O-Gen**	**Consensus values**
**Confidence**	100 million iterations x 5 runs (ESS > 30,000)	R^2^ = 0.71	-
**Divergence date for all *S*. Typhimurium 135@**	May 1996 [Jul 1986 - Nov 2001]	Mid 1997	1996-1997
**Divergence date for *S*. Typhimurium 135@ farm clone**	May 2004 [Sep 2002 - Mar 2005]	Early 2003	2003-2004
**Substitution rate variable site**^**-1**^ **day**^**-1**^	7.6 × 10^-6^ [3.2 – 12.5 × 10^-6^]	4.4 × 10^-6^	4.4-7.6 × 10^-6^
**Substitution rate ** **site**^**-1**^ **year**^**-1**^	1.2 × 10^-6^ [4.8 – 19 × 10^-7^]	6.7 × 10^-7^	6.7-12 × 10^-7^
**SNP year**^**-1**^	5 [2-8]	3	3-5

Divergence and mutation rate estimates [95% highest posterior density intervals] from BEAST and Path-O-Gen analyses, and consensus values across these two methods. ESS, effective sample size; R^2^, Pearson correlation between date of isolation and maximum likelihood tree branch length.

STm4 and STm10 were comparatively very distant from the farm clone, separated from it by 75–100 SNPs and sharing a much older mrca that diverged around 1996–1997 (95% HPD, 1986–2001) (Figure 2). This further supports that STm10 and STm4, while certainly related to the farm clone, cannot be considered part of the same chains of transmission that caused the Tasmanian outbreaks and did not derive from the *S*. Typhimurium 135@ population circulating at the farm. Consistent with this, STm4 and STm10 were isolated from sporadic cases of *S*. Typhimurium 135@ in Tasmania with no epidemiological links to the outbreaks, suggesting independent sources of *S*. Typhimurium 135@ infection.

While we cannot validate the divergence date estimates with independent data, MLVA analysis of our *S*. Typhimurium 135@ collection showed isolates with MLVA profiles identical to that of the outbreak clone (2-11-10-10-212) were detected in an Australian chicken (backyard, Queensland) and human infection (New South Wales) in 2004. This confirms the presence of the clone in Australia in 2004, consistent with predicted introduction of the ancestor into the Tasmanian chicken farm in 2003–2004. Interestingly, closely related STm135 isolates (MLVA profile 2-11-12-11-212) were collected during a Tasmanian gastroenteritis outbreak in 1994 and from a wild Tasmanian devil in 1996 (STm135, MLVA profile 2-10-7-10-212).

### Microevolution and natural selection within S. Typhimurium

Our data set provides a unique opportunity to examine the microevolutionary processes occurring in the *S*. Typhimurium population, within the spatiotemporal confines of a clonal bacterial population circulating in a host chicken population at a single farm over a 4–6 year period (beginning 2003–2005 and ending in 2008). We mapped each SNP onto the phylogenetic tree (numbers on each branch in Figure 2) and determined its effect on encoded proteins (synonymous SNPs, resulting in no amino acid changes; non-synonymous SNPs, resulting in amino acid changes; or SNPs in non-protein-coding regions), see Additional file 1: Table S1. Across all branches, the mean genome-wide rate of non-synonymous to synonymous substitutions (dN/dS) was 0.52. This is broadly indicative of purifying selection, suggesting that for every 2 non-synonymous SNPs that arise in the *S*. Typhimurium population, one is deleterious and is removed. As this selective process is expected to take time, a decline in dN/dS is sometimes observed when moving from recent time scales (dN/dS ~ 1, reflecting the underlying substitution rate without selection) through to longer time scales (dN/dS - > 0, as non-synonymous SNPs are removed over time) [27]. Because our data set includes isolates that are separated by varying amounts of evolution (within the farm clone; between the farm clone and other *S*. Typhimurium 135@; and between more diverse *S*. Typhimurium), we examined whether the dN/dS rate varied between branches associated with these different evolutionary scales. Among branches separating diverse lineages of Typhimurium (long-term evolution), the average dN/dS was 0.43. On branches separating the three clades of *S*. Typhimurium 135@ (STm4, STm10 and the farm clone; estimated above to encompass ~12 years of evolution), dN/dS values were 0.45, 0.48 and 0.45. Across all SNPs accumulating within the farm clone (estimated above to encompass 4–6 years of evolution), the dN/dS was 0.46. Hence there is no evidence that dN/dS declines through time in *S*. Typhimurium; rather, non-synonymous mutations appear to either be removed rapidly (within a few years, ~50% of non-synonymous SNPs) or become fixed within local subpopulations.

We have two outbreaks represented by multiple isolates, from which to examine the accumulation of SNPs during the course of an outbreak (Figure 2). We identified only 6 SNPs that arose during outbreaks; 5 of which were non-synonymous (Additional file 1: Table S1). This number is too small to draw conclusions from, but could be explained by underlying mutation rates without selection (assuming equal mutation rates, we would expect 3.2 non-synonymous mutations for every 1 synonymous mutation observed). For the other outbreaks, for which we have just one representative isolate each, it is impossible to distinguish SNPs that arose during the outbreak from those arising prior to the outbreak (i.e. during circulation at the farm). STm1 and STm2 were isolated at the farm, therefore their differences from the mrca reflect mutations that have arisen during circulation within the farm. The SNPs they have accumulated since the mrca show a dN/dS of 0.6, which is not significantly different from the average within the farm clone (0.46). There were also two internal branches within the clone that represent mutations arising in the farm; the branch leading to the second 2005 outbreak (Figure 2, 1 non-synonymous and 5 synonymous SNPs; dN/dS ~ 0.06) and the branch leading to the 2008 outbreak (Figure 2, 2 non-synonymous and 6 synonymous SNPs plus one intergenic SNP; dN/dS ~ 0.1). Given the short time scale associated with these branches (1–4 years), the lack of non-synonymous SNPs is suggestive of strong purifying selection, which might be expected in a large bacterial population competing within a small, geographically confined host population such as a farm. However since no such paucity of non-synonymous SNPs was evident in the farm isolates STm1 and STm2, and the mean dN/dS within the clone was the same as outside the clone, these low dN/dS values are probably anomalies and there is no evidence for significant difference in the short-term and long-term impacts of purifying selection within this *S*. Typhimurium population.

### Phage content in S. Typhimurium 135@

**Figure 3:**
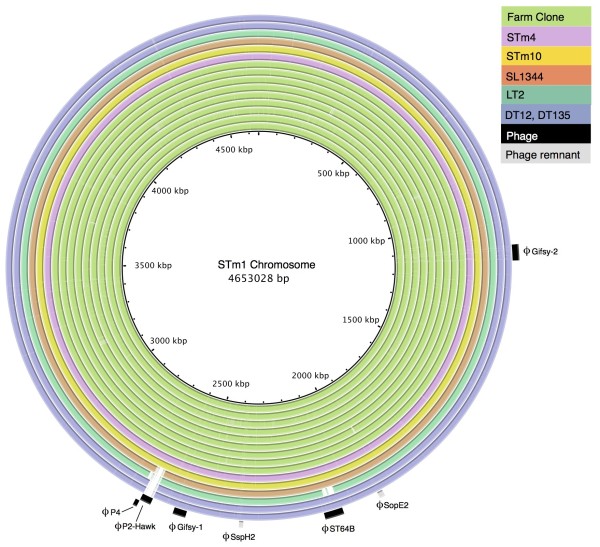
**Phage content variation among *S. *Typhimurium 135@, SL1344, LT2, DT135 and DT12.** BRIG diagram showing the STm1 chromosome as a reference; coloured rings indicate the coverage of STm1 sequences among contigs from other *S*. Typhimurium 135@ and the two reference chromosomes. The location of prophage sequences and phage remnants in the *S*. Typhimurium 135@ genome is indicated around the outside.

**Figure 4:**
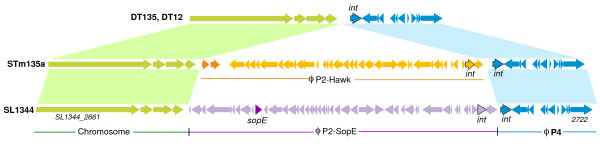
**Variable P2-P4 phage region in *S. *Typhimurium 135@ compared to SL1344, DT135 and DT12.** Orange arrows indicate genes encoded in the P2-Hawk prophage sequence from *S*. Typhimurium 135@; the two cargo genes are darkened. Purple arrows indicate genes encoded in the P2 SopEϕ phage from SL1344; the *sopE* effector gene is darkened. The P4 phage, conserved in SL1344, DT135 and *S*. Typhimurium 135@ is shown in blue. The integrase genes of each prophage are labelled (*int*).

We also investigated differences in gene content in *S*. Typhimurium 135@. There were very few differences in chromosomal gene content between *S*. Typhimurium 135@ and SL1344 (Figure 3). SL1344 contains five prophage sequences as well as prophage remnants, which were mainly conserved in all *S*. Typhimurium 135@ isolates (Figure 3). The P2 phage remnant of SL1344 (coordinates 2,815,301 - 2,825,986) was missing from *S*. Typhimurium 135@, and the Gifsy-1 phage of *S*. Typhimurium 135@ is much closer to that of DT2 than SL1344. The major difference in phage content between SL1344 and *S*. Typhimurium 135@ was the replacement of SL1344’s SopEϕ prophage with a novel prophage we have designated P2-Hawk (between bases 47,071-94,739 in contig 102, WGS project AMDX02). In SL1344, the P2 SopEϕ prophage is inserted in front of another prophage sequence, a 10 kbp P4 prophage located next to SL1344_2723 (Figure 4). The *S*. Typhimurium 135@ chromosomes have the same P4 phage located at the same site, but in place of the 34 kbp SopEϕ prophage there is a novel P2 prophage 32 kbp in size (Figure 4). This P2-P4 phage region was completely conserved among all the *S*. Typhimurium 135@ isolates (Figure 3). The novel P2-Hawk phage in the *S*. Typhimurium 135@ isolates carries two cargo genes – one is a hypothetical protein with no known function, however the second shares 70% amino acid sequence identity with a phage repressor protein (AbiC; Pfam14355) (dark orange, Figure 4). Nucleotide BLAST searches of the NCBI non-redundant database (accessed July 2013) identified prophage sequences with substantial sequence homology to P2-Hawk in *S.* Typhimurium T000240 and in ten other *S. enterica* serovars (Agona, Give, Hadar, Heidelberg, Johannesburg, Newport, Paratyphi A, Paratyphi C, Virchow and Weltevreden) as well as *S. enterica* subspecies *houtenae*, often at other insertion sites in the chromosome. These other prophages however lack the two cargo genes. The phage content of the DT135 and DT12 genomes were very similar to *S*. Typhimurium 135@ (Figure 3), except that they lacked a P2 phage near the P4 integration site (Figure 4) and DT12 harboured an additional P22 phage.

### Plasmid content in S. Typhimurium 135@

All *S*. Typhimurium 135@ isolates carried a copy of the *S*. Typhimurium virulence plasmid pSLT. We identified only two SNPs that separated the *S*. Typhimurium 135@ plasmids from SL1344’s pSLT (synonymous C- > T in codon 11 of hypothetical protein SL1344_P1_0060; synonymous G- > A in codon 94 of putative outer membrane protein SL1344_P1_0095) and a single SNP that separated the plasmids of the outbreak clone from those of STm4 and STm10 (non-synonymous G- > T in codon 49 of putative resolvase SL1344_P1_0062). The STm4 and STm12 plasmids also harboured one unique SNP each, both of which were non-synonymous (SL1344_P1_0091, SL1344_P1_0064). No differences in virulence plasmid gene content were identified, amongst the *S*. Typhimurium 135@ isolates or in comparison with pSLT.

**Figure 5:**
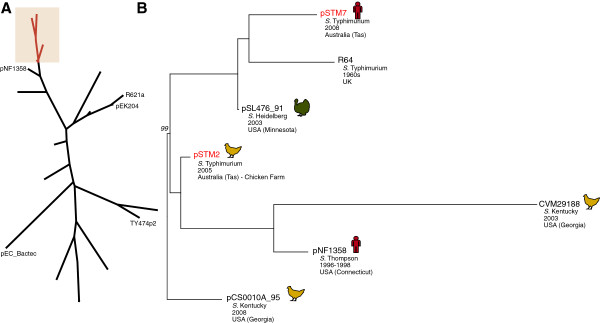
**Phylogenetic analysis of IncI1 plasmids identified in *S. *Typhimurium 135@. (A)** Maximum likelihood phylogeny of 23 publicly available IncI1 plasmids (listed in Table 4) together with pSTM2 and pSTM7 identified in this study. Key plasmids from these 23 are labeled. The shaded region indicates the clade containing pSTM2 and pSTM7, shown in detail in **(B)**. Bootstrap support for this clade is 88%. **(B)** Bootstrap values for each branch are 100%, except one node as indicated (99%).

Two of the *S.* Typhimurium 135@ isolates carried additional plasmids, both of the IncI1 incompatibility group. STm2 (farm isolate, 2005) carried pSTM2 (accession KF290378) and STm7 (human case, 2008) carried pSTM7 (accession KF290377) which included a *sul2* gene that confers resistance to sulfonamide antimicrobials. Plasmid pSTM2 did not have any antimicrobial resistance genes but contained a 4.8 kbp region which was not present in pSTM7, encoding aDNA adenine methytransferase (PSTM2_00004), a DNA damage inducible protein I (PSTM2_00006) and several hypothetical proteins (PSTM2_00001, PSTM2_00002, PSTM2_00003, PSTM2_00005). This is consistent with antimicrobial susceptibility typing, which showed that all 12 *S*. Typhimurium 135@ isolates were susceptible to all antimicrobials tested with the exception of STm7 which was resistant to sulphathiazole. To determine whether pSTM2 and pSTM7 could be related via conjugal transfer between *S*. Typhimurium 135@ host strains or via microevolution within the farm’s *S*. Typhimurium 135@ population, we constructed a phylogenetic tree of the IncI1 core genes for these and 23 publicly available IncI1 plasmids (Table 4, Figure 5). This analysis showed that pSTM2 and pSTM7 belong to a subclade including 5 other IncI1 plasmids isolated from *Salmonella enterica*, three of which were also associated with poultry (Figure 5). However the tree indicates pSTM2 and pSTM7 cannot be directly related to one another via transfer or local microevolution (Figure 5); rather, they represent distinct transfers of IncI1 plasmids into members of the *S*. Typhimurium 135@ farm clone. This is also supported by plasmid MLST analysis (Table 4), which shows pSTM2 (ST7) and pSTM7 (ST3) belong to different clonal complexes identified via analysis of much larger plasmid collections (clonal complex 7, clonal complex 3). Interestingly STm6, isolated from the restaurant to which the STm7 case was linked, did not carry an IncI1 plasmid, nor did STm12 which was isolated from another human case from the same outbreak. Hence plasmid pSTM7 is likely to have been acquired *in vivo* during the infection in the human host rather than in the farm environment; this could potentially be related to selection for the *sul2* resistance gene in pSTM7.

**Table 4:** IncI1 plasmid sequences analysed in this study

**Plasmid**	**Host bacteria**	**Source**	**MLST**	**Accession**	**Reference**
P9	*Shigella sonnei*	-	ST11	AB021078	[28]
R64	*Salmonella* Typhimurium	-	ST13	AP005147	[29]
pSE11-1	*Escherichia coli*	Human	ST14	AP009241	[30]
R621a	*Salmonella* Typhimurium	-	Novel	AP011954	[31]
SL476	*Salmonella* Heidelberg	Turkey	ST15	CP001118	[32]
CVM29188	*Salmonella* Kentucky	Chicken	ST12	CP001121	[33]
pRK1	*Escherichia coli*	Soil	Novel	CP002186	[34]
TY474p2	*Salmonella* Typhimurium	-	ST27	CP002489	[22]
pEKO1101	*Escherichia coli*	Human	Novel	CP002517	[35]
pUKMNK88_91	*Escherichia coli*	Pork	ST19	CP002731	[36]
pESBL-EA11	*Escherichia coli*	Human	ST31	CP003290	[37]
pEK204	*Escherichia coli*	Human	ST16	EU935740	[38]
p746	*Escherichia coli*	Human	Novel	FN822748	[39]
pEC_Bactec	*Escherichia coli*	Horse	ST31	GU371927	[40]
pKHSB1	*Shigella sonnei*	-	ST16	HF572032	[41]
pND11_107	*Escherichia coli*	Human	ST69	HQ114281	[42]
pND12_96	*Escherichia coli*	Human	ST19	HQ114282	[42]
pCS0010A_95	*Salmonella* Kentucky	Chicken	ST7	HQ114283	[42]
pSH1148_107	*Salmonella* Heidelberg	-	ST26	JN983049	[43]
Plm	*Escherichia coli*	-	Novel	JQ901381	[44]
pSD107	*Salmonella* Derby	Pork	ST26	JX566770	[45]
pNF1358	*Salmonella* Thompson	Human	ST11	NC_019011	[46]
pSTM2	*Salmonella* Typhimurium	Farm	ST7	KF290378	This study
pSTM7	*Salmonella* Typhimurium	Human	ST3	KF290377	This study

## Discussion

### Implications for understanding whole genome sequencing (WGS) data in the context of outbreak investigation and source attribution

It is important to note that both the epidemiological data and the phylogenetic approach are crucial for proper interpretation of the data. Firstly, the epidemiological investigation was crucial in linking the infections to specific food sources and tracing these back to a single farm, with a high degree of confidence. This enables us to interpret the clonal group (red branches in Figures 1, 2) as representing a diverse bacterial source population at the farm; without the epidemiological information and farm isolates we could only guess at a common source. Secondly, phylogenetic inference is critical to reaching this understanding. If we were to consider only pairwise differences between isolates, we would be able to identify close relationships between isolates from the same outbreak (0–5 SNPs) and conclude these form part of direct transmission chains; but we would not be able to resolve the nature of the relationships between different outbreaks or between farm isolates. However, the phylogenetic inference shows us that all the outbreaks and the farm isolates share a recent common ancestor, which enables us to understand that these isolates form a single local population resulting from just a few years of clonal expansion. Other studies have also been able to demonstrate the benefits of using WGS and phylogenetic inference in addition to epidemiology in outbreak investigations, finding that WGS methods are stable and consistent with epidemiological results [47,48].

Our study provides insight into the level of diversity that can be expected during *Salmonella* outbreaks. It highlights that when considering whether a specific food product, implicated by epidemiological investigation of outbreak cases, is in “immediate source” involved directly in disease transmission (in this case the bakery piping bag and restaurant aioli), we should expect very few mutations (0–1 SNPs) between bacteria isolated from the proposed transmission vehicle and those from cases. However when tracing these food products back to a potential “ultimate source” (in this case a farm), we must understand that we are likely to be sampling from a bacterial source population which has diversified to some extent, and that the transmission chain that led to human infections may represent just one sublineage of the overall diversity present in the ultimate source population. We therefore need to expect more variation between infection isolates and isolates from potential ultimate sources, without ruling out a direct transmission link. Our data also suggests that, if multiple isolates are obtained from a suspected source population, there will likely be added value in generating WGS data on many or all of them rather than sequencing a representative isolate. Even if no source isolates are identical to infection isolates, establishing the diversity range of possible ancestors of source and infection isolates will likely be informative, as it was in this case.

Our data suggests that, as long as epidemiological and phylogenetic approaches were combined as they were here, most of the conclusions from this retrospective analysis could have been made during the outbreak investigation. If WGS had been performed prospectively during the outbreaks, it would have confirmed the existence of a close transmission chain between case (STm5) and food source (STm3) in outbreak 2 (1 SNP), and a close relationship between outbreaks 1 and 2 (10 SNPs, recent common ancestor), see Figure 2. WGS would have confirmed immediately that STm10 (outbreak 3) was not related to the contemporaneous outbreaks 1 and 2 (>75 SNPs) but that outbreak 5 probably was (Figure 2). When the farm isolates were obtained soon after, WGS would have confirmed that these also derived from the common ancestor of outbreaks 1, 2 and 5 (Figure 2), lending further weight to the conclusions of the epidemiological investigation by providing strong phylogenetic evidence that outbreaks 1, 2 and 5 stemmed from a common source population at the farm.

### Purifying selection within S. Typhimurium

We found that genome-wide dN/dS was approximately 0.5 across all branches of the *S*. Typhimurium tree. This is consistent with a previous estimate based on comparison of two *S*. Typhimurium lineages (0.53) [18]. Our observation that dN/dS rapidly reaches this level and then remains consistent across the phylogeny, is strikingly similar to the pattern observed in *S. enterica* serovar Typhi, the agent of typhoid fever [49]. In *S*. Typhi, dN/dS within WGS-defined subclones was roughly equal to one, reflecting the underlying mutation rates and an absence of purifying selection over short time scales; this is very similar to our observation that intra-outbreak SNPs in *S*. Typhimurium were consistent with underlying mutation rates. Similarly, in *S*. Typhi SNPs that differentiated WGS-defined lineages (similar to the scale of the farm clone vs STm4 and STm10) or occurred on the oldest internal branches of the WGS tree (similar to the scale of the non-*S*. Typhimurium 135@ branches in our *S*. Typhimurium tree) showed a dN/dS 0.46-0.52 [49]. This suggests that in both *S*. Typhi and *S*. Typhimurium, approximately half of all non-synonymous SNPs are somewhat deleterious and removed rapidly from the population via purifying selection; however after this there is very little evidence of further selection. A recent study of *S. enterica* serovar Agona found a similar dN/dS rate of 0.67, and no evidence of adaptive selection within the *S*. Agona population [50].

### Substitution rates in S. Typhimurium

We estimated a substitution rate of 6.7-12 × 10^-7^ substitutions site^-1^ year^-1^ or 3–5 SNPs chromosome^-1^ year^-1^ among the *S*. Typhimurium 135@ isolates. We did not perform this analysis for the whole *S*. Typhimurium data set as there was no relationship between branch lengths and date of isolation across the rest of the tree. Our rate is faster than that estimated within two lineages of *S*. Typhimurium causing invasive typhoid-like disease in Africa (1.9 × 10^-7^ and 3.9 × 10^-7^ substitutions site^-1^ year^-1^ or 1–2 SNPs chromosome^-1^ year^-1^), which were calculated using methods similar to our whole-genome SNP analysis with BEAST [26]. This may be because our analysis reflects short term evolution (isolates collected over 4 year period, with estimated mrca in 1996 reflecting 12 years of evolution) in a small and spatiotemporally contained population (mostly within a single farm host population), whereas the African population analysis reflects longer term evolution (isolates collected over a 22 year period, mrca in 1960 reflecting 50 years of evolution) in a larger host population. The recent *S.* Agona population genomics analysis estimated a much lower substitution rate of 5.7 × 10^-8^-1.3 × 10^-7^ site^-1^ year^-1^, however this analysis included multiple different lineages and displayed substantial variation in substitution rates across the phylogeny, hence it is not directly comparable [50].

### Phage and plasmid variation

The genomes of *S*. Typhimurium SL1344 and *S*. Typhimurium 135@ share a P4 prophage sequence, with different P2 prophages adjacent to it (Figure 4). A P2 prophage (Fels-1) exists at the same location in *S*. Typhimurium LT2, but no P4 phage is present. P4 is a defective phage that lacks its own genes for capsid, tail and lysis functions. Instead it utilises P2 as a helper phage to build structural components and package its DNA [51]. There is no evidence that physical proximity of the P2 and P4 prophages is important for this interaction, so the close physical link between P2 and P4 prophages at this locus in different *S*. Typhimurium chromosomes may be related to preferred integration sites rather than the functional interaction between phages. Sequenced *S.* Tyhimurium isolates DT135, DT12 and LT12 [26] have the same P4 prophage as SL1344 and *S*. Typhimurium 135@, but without accompanying P2 prophage. The P2 phage in SL1344 encodes SopE, a type three secreted effector protein associated with host cell invasion. A recent screen of SL1344 transposon mutants in mouse, chicken, calf and pig colonization models [52] showed that SopE mutants were attenuated in their ability to colonize the gut of chickens, calves and pigs (data available at http://www-tradis.vet.cam.ac.uk). No data on SopE mutants was obtained for the mouse infection model so the importance of SopE in mammalian infection or gut colonization is unclear. However the ability of the SopE-negative *S*. Typhimurium 135@ to circulate in the chicken farm for many years, and to cause several large outbreaks of gastroenteritis in humans, indicates that it is not critical for colonization of chickens or for virulence in humans. This may be due to the redundancy of secreted effectors in the *S*. Typhimurium genome - the SopE2 protein, conserved in *S*. Typhimurium 135@, is 70% identical at the amino acid level to the phage-encoded SopE protein. The P2-Hawk phage in *S*. Typhimurium 135@ carried two cargo genes, which are not found in homologous phages in other *S. enterica*. One of these genes has close homology to a protein associated with conferring resistance to specific phages in *Lactococcus lactis*[53]. It is intriguing to speculate whether these genes may also confer resistance to certain *Salmonella* phages, or even help the P2-Hawk phage to repress the activity the P4 phage and prevent it from hijacking the P2 machinery to assist with its own dissemination.

The SNPs identified within the *S*. Typhimurium virulence plasmid pSLT were entirely compatible with the chromosomal SNP phylogeny, indicating no evidence for transfer of the plasmid between members of the host bacterial population. The difference between and farm clone and sporadic isolates in the repeat copy numbers for the STTR10 VNTR, located in the pSLT plasmid, may be an indication that this VNTR is relatively stable and further supports that the pSLT plasmid is stable within the clone. The detection of two independent acquisitions of IncI1 plasmids, pSTM2 and pSTM7, in the *S*. Typhimurium 135@ population is interesting. According to the IncI1 plasmid MLST scheme, pSTM2 and pSTM7 belong to clonal complexes 7 and 3, respectively. Both are associated with *S. enterica* and *E. coli* isolated from humans, poultry and occasionally other food animals (see e.g. [54]), and frequently carry beta-lactamase CTX-M genes, encoding resistance to third generation cephalosporins (http://pubmlst.org/plasmid/, accessed June 2013 [55]). The novel plasmids pSTM2 and pSTM7, which lack CTX-M genes, may be useful comparators to investigate the emergence of CTX-M and other resistance genes in related IncI1 plasmids.

## Conclusions

We have shown that *S*. Typhimurium 135@ is closely related to the well-studied laboratory strain SL1344, with very few differences in terms of SNPs or gene content. However we identified several minor phage differences which could account for the phenotypic differences in phage type, most notably the novel prophage P2-Hawk replacing the SopEϕ prophage of SL1344. We also identified two novel IncI1 plasmids in *S*. Typhimurium 135@, which belong to plasmid lineages that are associated with *Enterobacteriaceae* in poultry in many other parts of the world. By analysing the genomes of *S*. Typhimurium 135@ from a series of outbreaks, we obtained estimates of short-term mutation rates and population structure in this important foodborne pathogen, which will be useful in interpreting genomic data in future outbreak investigations.

## Methods

### Sequencing and assembly

Genomic DNA was extracted from fresh overnight subcultures of *S*. Typhimurium 135@ isolates using QIAamp DNA Mini Kit and QIAcube (Qiagen) and transferred to the Australian Genome Research Facility (AGRF) for multiplex sequencing on Illumina HiSeq (11 isolates multiplexed in one lane), generating 100 bp paired-end reads. STm5 was sequenced earlier in a single lane of HiSeq generating 36 bp paired-end reads. Illumina reads were assembled using SPAdes 2.4.0 [56], resulting in a median of 118 contigs per genome (range, 84–458 contigs), covering a median of 4.94 Mbp of sequence (range, 4.93 – 5.04 Mbp), with N50 of 21 kbp - 259 kbp and mean read depth 300× - 1000×. Read mapping to the available finished *S*. Typhimurium reference chromosome sequences (Table 2) using BWA0.7.5a [57] revealed the closest reference for all *S*. Typhimurium 135@ isolates was *S*. Typhimurium SL1344 (phage type DT44, accession NC_016810.1). Each set of contigs was ordered against the *S*. Typhimurium SL1344 chromosome and plasmid using MUMmer 3.23 [58] and ABACAS (version 1.3.1, http://abacas.sourceforge.net/) and annotated using NCBI’s PGAP (http://www.ncbi.nlm.nih.gov/genome/annotation_prok/). Prophage sequences were identified with the help of PHAST [59] and comparison to the finished genomes of *S.* Typhimurium SL1344 (NC_016810.1) and LT2 (NC_003197.1) was performed using ACT (version 12.0.0) [60].

### Phylogenetic and evolutionary analysis

SNPs were identified by mapping reads to a reference sequence using BWA [57] and calling SNPs with SamTools 0.1.18 [61]. Raw SNP calls were filtered for quality (phred score ≥20), depth (≥10x) and homozygosity as in [62]. References used were: (i) chromosome – *S*. Typhimurium SL1344 (NC_016810.1), (ii) virulence plasmid - *S*. Typhimurium SL1344 plasmid pSLT (NC_017720.1), and (iii) IncI1 plasmid – *S*. Thompson plasmid pNF1358 (NC_019011.1). Other publicly available genome sequences were included in phylogenies by first simulating 1 million 100 bp paired end reads from the finished sequence, and mapping these to the reference sequence in the same manner as the Illumina reads. SNP calls located in repeat regions, insertion sequences or phage sequences were excluded from phylogenetic analysis (391 kbp or 8% of the SL1344 chromosome). Maximum likelihood phylogenetic trees were inferred using RAxML 7.2.8 [63] to analyse the concatenated alignment of SNP alleles (GTR + Γ model of nucleotide substitution, 10 replicate runs and 1,000 bootstraps). The neighbour-joining split network (Figure 1) was inferred from the same alignment using SplitsTree4 [64]. SNPs were mapped onto the trees using the *baseml* function of the PAML software package (version 4.7) [65]. dN/dS was approximated by dividing the ratio of non-synonymous SNPs to synonymous SNPs by the ratio of possible non-synonymous and synonymous mutations across all protein coding sequences in the SL1344 reference genome, which we calculated to be 3.2 using the codon usage function (*cusp*) in the EMBOSS package (version 6.6.0) [66].

### Divergence dating

Isolation dates were converted to a continuous discrete variable representing days since 1900. Path-O-Gen v1.3 was used to analyse the association between this variable and branch lengths in the rooted maximum likelihood tree for *S*. Typhimurium 135@ (Figure 2). For BEAST analysis (v1.6), we used the same isolation date variable (expressed in days since 1900) and the same concatenated SNP alignment as used for RAxML analysis. We investigated 4 alternative models resulting from the combination of two demographic models (constant population size and Bayesian Skyline) and two molecular clock models (strict and relaxed lognormal). For each model, we ran 5 replicate runs for 100 million iterations, and combined the results after excluding the first 10 million iterations as burn-in. The relaxed clock models estimated an uncorrelated standard deviation of the mutation rate (ucld.stdev) abutting zero, providing evidence for a strict rather than relaxed molecular clock. The two demographic models gave nearly identical results (log Bayes Factor = 0.16) and the Bayesian Skyline plot indicated a constant population size. Hence the results reported in Table 3 are those from the strict clock, constant population size model only. Since our estimates are based on a SNP alignment with time expressed in days, the raw estimates were in units of substitutions per variable site per day. These were scaled to genome-wide units of substitutions per site per year by multiplying the estimates by constant *k* = *n*/*N* × 365 where *n* is the number of SNP sites in the alignment (1,871), N is the total positions considered for SNP calling (4,487,272); hence *k* = 0.1522.

### Gene content analysis

The phage sequences of *S*. Typhimurium 135@, SL1344 and LT2 were identified with the help of PHAST [59] and the genome annotations for SL1344 and LT2. Pairwise comparisons were examined using ACT [60] and multiple genome comparisons were visualised using BRIG (version 0.95) [67]. To identify contigs belonging to the virulence plasmid pSLT (accession NC_017720), contigs which did not map to SL1344 with ABACAS/MUMmer were compared to pSLT using ABACAS/MUMmer and nucleotide BLAST. The comparison of non-chromosomal contigs to pSLT was visualised using ACT. Contigs not mapping to the chromosome or pSLT were identified in STm2 and STm7, these were then used as blastn queries to the NCBI nr database to investigate their origin. This showed that both the STm2 and STm7 contigs had close homology *S. enterica* IncI1 plasmid pNF1358 (accession NC_019099). We therefore named these contigs pSTM2 and pSTM7 respectively, and compared them to the pNF1358 sequence using ACT to identify regions of difference and confirm that they represent complete IncI1 circular replicons. These were then isolated using Prokka (version 1.5.2, http://vicbioinformatics.com/) and submitted to GenBank. Phylogenetic inference is described above, with core genes defined as genes that were present in all plasmid sequences. Plasmid MLST for all IncI1 plasmids was determined using SRST [68].

### Antimicrobial susceptibility and multi-locus VNTR analysis (MLVA)

The sequenced *S*. Typhimurium 135@ isolates were tested for resistance to the following antimicrobials (MIC cut-off for resistance): ampicillin (>16 μg/ml), streptomycin (>8 μg/ml), tetracycline (>8 μg/ml), chloramphenicol (>16 μg/ml), sulphathiazole (>512 μg/ml), trimethoprim (>8 μg/ml), kanamycin (>32 μg/ml), nalidixic acid (>16 μg/ml), spectinomycin (>50 μg/ml), gentamicin (>8 μg/ml), ciprofloxacin (>0.06 μg/ml) and cefotaxime (>1 μg/ml). MLVA profiles for 1,930 *S.* Typhimurium 135@ were checked for similarity to the outbreak isolates by the Microbiological Diagnostic Unit Public Health Laboratory (MDU PHL), Victoria. MLVA profiles were generated using a multiplex assay targeting five VNTR loci [69]. The resulting profiles are expressed here in the form of repeat copy numbers for locus STTR9, STTR5, STTR6 and STTR10; and an allele code for STTR3, using the methods and nomenclature of [70]. Hence the profile 2-11-10-10-212 indicates 2 repeats at locus STTR9, 11 repeats at STTR5, 10 repeats at STTR6, 10 repeats at STTR10 and the 212 allele of locus STTR3 (corresponding to 524 bp at this locus).

### Sequence data accessions

The annotated *S.* Typhimurium 135@ whole genome sequences were deposited as Whole Genome Shotgun projects at DDBJ/EMBL/GenBank and Illumina reads are available in the NCBI short read archive, accessions are given in Table 1. The plasmids pSTM2 and pSTM7 were deposited in GenBank under accessions KF290378 and KF290377, respectively.

## Competing interests

The authors declare that they have no competing interests.

## Authors’ contributions

JH analysed data and helped to draft the manuscript. DJE performed mapping and SNP calling analysis. KD performed MLVA analysis and extracted DNA for sequencing. LH analysed the prophage regions. HBJ contributed to data interpretation and manuscript writing. GH suggested the cluster be studied, participated in the design and coordination of the study and provided data for analysis. KEH conceived the study, performed data analysis and drafted the manuscript. All authors read and approved the final manuscript.

## Supplementary Material

Additional file 1: Table S1 Details of SNPs that define, or vary within, the *S*. Typhimurium 135@ group. Click here for file
